# The transcription factor 7-like 2 (*TCF7L2*) polymorphism may be associated with focal arteriolar narrowing in Caucasians with hypertension or without diabetes: the ARIC Study

**DOI:** 10.1186/1472-6823-10-9

**Published:** 2010-05-17

**Authors:** Yu Yan, Ronald Klein, Gerardo Heiss, Cynthia J Girman, Ethan M Lange, Barbara E Klein, Kathryn M Rose, Eric Boerwinkle, James S Pankow, Frederick L Brancati, Christie M Ballantyne, Anna Köttgen, Kari E North

**Affiliations:** 1 Department of Epidemiology, University of North Carolina, Chapel Hill, NC, USA; 2 Department of Ophthalmology and Visual Sciences, University of Wisconsin, Madison, WI, USA; 3 Department of Epidemiology, Merck Research Laboratories, West Point, PA, USA; 4 Department of Genetics, University of North Carolina, Chapel Hill, NC, USA; 5 Human Genetics Center, University of Texas Health Science Center, Houston, TX, USA; 6 Division of Epidemiology and Community Health, University of Minnesota, Minneapolis, MN, USA; 7 Department of Epidemiology, Johns Hopkins Bloomberg School of Public Health, Baltimore, MD, USA; 8 Department of Medicine, Johns Hopkins University, Baltimore, MD, USA; 9 Department of Medicine, Atherosclerosis and Vascular Medicine, Baylor College of Medicine, Houston TX, USA; 10 Carolina Center for Genome Sciences, University of North Carolina, Chapel Hill, NC, USA

## Abstract

**Background:**

Transcription factor 7-like 2 (*TCF7L2*) has emerged as a consistently replicated susceptibility gene for type 2 diabetes, however, whether the *TCF7L2 *gene also has similar effects on the retinal microvasculature is less clear. We therefore aimed to investigate the association between the transcription factor 7-like 2 (*TCF7L2*) rs7903146 polymorphism and retinal microvascular phenotypes in the Atherosclerosis Risk in Communities (ARIC) Study (1993-1995).

**Methods:**

This was a population-based, cross-sectional study of 10,320 middle-aged African American (n = 2,199) and Caucasian (n = 8,121) men and women selected from four United States communities to examine the association between *TCF7L2 *rs7903146 polymorphism and retinal microvascular signs (retinopathy, focal arteriolar narrowing, arteriovenous nicking, arteriolar and venular calibers). Photographs on one randomly selected eye were graded for presence of retinal microvascular signs and used to measure retinal vessel calibres.

**Results:**

After adjusting for age, sex, study center, mean arterial blood pressure, total serum cholesterol, triglycerides, and other covariates, few associations of *TCF7L2 *rs7903146 and retinal microvascular signs were noted. *TCF7L2 *rs7903146 T risk allele was significantly associated with focal arteriolar narrowing in Caucasians with hypertension [odds ratio (OR)_CT vs. CC _(95% CI) = 1.25 (1.09-1.44); OR_TT vs. CC _= 1.56 (1.18-2.06); *P *= 0.002] and in Caucasians without diabetes [OR _CT vs. CC _= 1.18 (1.06-1.32); OR _TT vs. CC _= 1.40 (1.12, 1.75); *P *= 0.003]. No significant association of the *TCF7L2 *rs7903146 polymorphism and retinal vascular signs was noted among African American individuals.

**Conclusions:**

*TCF7L2 *rs7903146 is not consistently associated with retinal microvascular signs. However, we report an association between the *TCF7L2 *rs7903146 polymorphism and focal arteriolar narrowing in Caucasians with hypertension or without diabetes. Further research in other large, population-based studies is needed to replicate these findings.

## Background

Retinal microvascular signs (e.g. retinopathy) and changes in retinal vessel caliber are common fundus findings in adults aged 40 years and older [1]. Narrowing in retinal vascular caliber has been shown to predict the risk of diabetes [2] and to be related to retinopathy in people with diabetes [3], hypertension, or cardiovascular disease in the general population [1]. In addition to risk factors such as diabetes and hypertension, genetic factors may also play a role in the development of these retinal microvascular signs [4,5].

Transcription factor 7-like 2 (*TCF7L2*), a Wingless and Int (Wnt) signaling-associated transcription factor located on chromosome 10q25, has emerged as a consistently replicated susceptibility gene for type 2 diabetes [6-8], possibly through the impairment of glucagon-like peptide-1-induced insulin secretion [9]. In our previous work, we confirmed that the T allele at single nucleotide polymorphism (SNP) rs7903146 located in intron 3 of *TCF7L2 *confers risk for incident type 2 diabetes in middle-aged African Americans and Caucasians [7]. However, whether the *TCF7L2 *gene also has similar effects on the retinal microvasculature is less clear. To our knowledge, no studies examining the association of the *TCF7L2 *gene to retinal microvascular signs have been conducted but two studies evaluated retinopathy, which relies on less precise global assessments or self-report.

A study in a French population reported no evidence of an association with prevalent, severe diabetic retinopathy [10], whereas the InCHIANTI study indicated an association of the *TCF7L2 *gene with reported diabetic retinopathy [11], although the estimates were notably imprecise. Moreover, potential effects of hypertension on the association of *TCF7L2 *gene and retinopathy have been largely unexplored.

In this study, we investigated whether the *TCF7L2 *rs7903146 polymorphism is associated with retinal microvascular signs and retinal vessel caliber in a large community-based cohort of African-American and Caucasian middle-aged adults. A second objective is to evaluate whether the effect of the rs7903146 SNP varies by hypertension or diabetes status.

## Methods

### Study population

The ARIC Study is an ongoing, longitudinal cohort study of cardiovascular and other major diseases among 15,792 men and women, aged 45 to 64 years old at baseline (1987-1989), selected from 4 US communities: Forsyth County, NC; Jackson, MS; the northwestern suburbs of Minneapolis, MN; and Washington County, MD [12]. By design, African-Americans were over-sampled at the Forsyth County site and were exclusively sampled in Jackson and thus constituted 27% of the baseline cohort. Of the 15,792 participants at baseline, 12,887 (86%) returned for the third examination when retinal photography was first performed in 1993-1995.

We excluded ARIC participants who were not African-American or Caucasian (*n *= 38), African-Americans from Minnesota and Maryland field centers (*n *= 42), participants with missing genotype data or who did not provide consent for the use of their DNA (*n *= 803), participants who did not have retinal photographs (*n *= 224), participants who had ungradeable photographs (*n *= 1458), and participants who had diabetes diagnosed before 20 years old (*n *= 2). After these exclusions, 10,320 participants (2,199 African American and 8,121 Caucasians) were available for analysis. Characteristics of participants with and without gradable retinal photographs have been previously described [13,14]. The institutional review boards at all participating institutions and at the Fundus Photograph Reading Center at the University of Wisconsin approved the procedures and all participants included in the analysis gave informed consent.

### Assessment of Retinal Microvascular Signs

The retinal photography procedures and grading of retinal microvascular signs have been published in detail elsewhere [13]. In brief, one eye was randomly selected from each participant and a 45° retinal photograph, centered on the region of the optic disc and the macula, was taken using an autofocus film camera after a five-minute dark adaptation. If the selected eye was considered too difficult or not possible to photograph with adequate quality, the other eye was photographed instead.

These retinal photographs were evaluated at the Fundus Photograph Reading Center at the University of Wisconsin, Madison, by trained graders who were masked to participant characteristics. We measured and defined the presence of focal retinal microvascular abnormalities, including retinopathy, arteriovenous (AV) nicking, and focal arteriolar narrowing. Retinopathy was defined based on the presence of any of the following lesions: retinal hemorrhages (blot or flame shaped), microaneurysms, soft or hard exudates, macular edema, intraretinal microvascular abnormalities, venous beading, swelling, or laser photocoagulation scars. AV nicking and focal arteriolar narrowing were defined as present if graded as definite or probable and as absent if not. Retinal arteriolar and venular calibers were measured using a computer-assisted approach. The fundus photographs were digitized and the diameters of all arterioles and venules in an area half to one disc diameters from the optic disc were measured. These diameters were summarized as the central retinal artery equivalent (CRAE) and the central retinal venular equivalent (CRVE) [13]. Quality control procedures have been previously reported [13].

### TCF7L2 Genotyping

The *TCF7L2 *rs7903146 SNP was genotyped by the ARIC Central Laboratory using Taqman^® ^assays (Applied Biosystems, Foster City, CA). Laboratory-designed probes were obtained from Applied Bioystems and primers from IDT (Coralville, IA). All PCR reactions took place in optical 384-well reaction plates (Applied Biosystems). Five percent of samples were re-genotyped for quality control as blind duplicates. The percent agreement between blind duplicates was 98% and the simple Kappa coefficient was 0.97 indicating good genotyping quality.

### Measurement of Covariates

Self-reported race, sex, and study center were ascertained at baseline (1987-1989). Other covariates including age, current smoking, obesity, total serum cholesterol, total serum triglycerides, mean arterial blood pressure, and antihypertensive medication were obtained at visit 3 (1993-1995). At each visit, blood pressure was measured three times using a random zero sphygmomanometer and the average of the last two measurements was used for analyses. Hypertension was defined as systolic blood pressure ≥140 mmHg or diastolic blood pressure ≥90 mmHg or current use of anti-hypertension medication use at visit 1, 2, or 3 [15]. Mean arterial blood pressure was defined as one-third of systolic blood pressure plus two-thirds of diastolic blood pressure at visit 3 [16]. Diabetes was defined as fasting serum glucose levels of at least 7.0 mmol/L (126 mg/dl), nonfasting glucose levels of at least 11.1 mmol/L (200 mg/dl), current use of hyperglycemic medications, or a self-reported physician diagnosis of diabetes at visit 1, 2 or 3 [17]. Plasma total cholesterol and triglyceride levels were measured by enzymatic methods; high-density lipoprotein cholesterol (HDL-C) was measured after dextran-magnesium precipitation of the non-HDL-C; and glucose was assessed by a modified hexokinase/glucose-6-phosphate dehydrogenase procedure [18]. Self-reported cigarette smoking exposure was defined as current smoking versus non-smoking obtained by a personal interview. Body mass index (BMI) was calculated as measured weight (kg) divided by the square of measured height (m^2^). Individuals with a BMI ≥30 kg/m^2 ^were classified as obese [19].

### Statistical Analysis

All analyses were stratified by race to crudely account for population stratification. To assess whether genotype distribution within each race departed from Hardy-Weinberg equilibrium, a *χ*^2 ^goodness-of-fit test was used. Logistic regression was used to model the association of focal retinal lesions (retinopathy, focal arteriolar narrowing, AV nicking) with the *TCF7L2 *rs7903146 polymorphism, and odds ratios (ORs) and 95% confidence intervals (CIs) were obtained. Following published literature [8] and our previous findings [7], we assumed an additive mode of inheritance and compared heterozygous CT-genotype and homozygous TT-genotype individuals to CC-genotype individuals, using the rs7903146 CC-genotype as the referent group. A variable taking on the values 0 for genotype CC, 1 for genotype CT, and 2 for genotype TT was used to test for log additive genetic effects in logistic regression models. Generalized linear models were used to obtain adjusted mean retinal vascular calibers (CRAE, CRVE) for each genotype of rs7903146. All models were adjusted for age, study center, sex, current smoking (yes/no), obesity (yes/no), total serum cholesterol, total serum triglycerides, mean arterial blood pressure, and antihypertensive medication. Hypertension was also included in the model when it was not assessed as an effect measure modifier.

As hypertension and diabetes are strongly associated with retinal microvascular signs and *TCF7L2 *is a diabetes-related gene, we assessed the potential interactions between genotype and hypertension, and interactions between genotype and diabetes on retinal microvascular phenotypes, respectively, and performed sub-group analyses with and without hypertension/diabetes. With regard to multiplicative interaction, variables were considered to be potential effect measure modifiers if they departed from multiplicativity assessed by the Wald *χ*^2 ^test for significance of the estimated β-coefficient for the interaction term [20]. For additive interaction, variables were considered as potential modifiers if departure from additivity was detected by the interaction contrast ratio (ICR) [20,21]. ICRs were quantified as follows: ICR = OR_AB - OR_A - OR_B + 1, where OR_AB represents the joint effect of hypertension/diabetes and the SNP, and OR_A and OR_B represent the main effects of hypertension/diabetes and the SNP, respectively [20]. Thus, ICR refers to the increased odds due to an additive interaction between hypertension/diabetes and the T risk allele adjusted for aforementioned covariates. Assuming an additive mode of inheritance, the ICR comparing TT to CT is equal to the ICR comparing CT to CC, thus only one ICR was reported. Departures from zero suggest that hypertension/diabetes and the SNP interact to cause retinal microvascular signs. The OR and the variance covariance matrix were used to calculate ICR values and *P *values [21]. For retinal vascular calibers (CRAE, CRVE), only the *P *value from multiplicative interaction test was estimated. A *p *value <0.05 was considered to indicate an important modifier for both multiplicative and additive interaction assessments, despite the multiple tests as interaction tests tend to be underpowered [22].

## Results

The rs7903146 T allele was observed with same frequency (29%) in African-American and Caucasian individuals, and the genotype frequencies for rs7903146 in both races were consistent with Hardy-Weinberg equilibrium (*P *> 0.05). Selected characteristics of the ARIC Study participants by race and genotype status are presented in Table 1. No statistically significant differences in demographic or behavioral characteristics (sex, and current smoking) were noted by genotype status except for age (P = 0.03) and HDL-C levels (P = 0.05) in Caucasian participants (Table 1). Moreover, no statistically significant differences in hypertension, mean arterial blood pressure, obesity, triglycerides, LDL-C, and total cholesterol by genotype were noted except for individuals with T allele who had significantly higher fasting glucose and were more likely to be diabetic in Caucasians (Table 1).

**Table 1:** Distribution of selected characteristics by race and rs7903146 genotype status

	African American				Caucasian			
		
	CC	CT	TT	***P *value**^**a**^	CC	CT	TT	***P *value**^**a**^
*n*	1099	923	177		4105	3321	695	

Age, years	58.4 ± 5.6	58.3 ± 5.4	58.9 ± 5.6	0.36	60.1 ± 5.6	59.9 ± 5.6	59.6 ± 5.6	0.03

Male sex	399 (36.31)	353 (38.24)	65 (36.72)	0.66	1894 (46.14)	1528 (46.01)	339 (48.78)	0.39

Current smoker	232 (21.28)	194 (21.20)	34 (19.32)	0.86	672 (16.38)	570 (17.17)	117 (16.83)	0.66

Obesity Present^b^	516 (46.95)	418 (45.29)	86 (48.86)	0.60	1223 (29.81)	926 (27.92)	200 (28.78)	0.20

Hypertension Present^c^	720 (65.51)	619 (67.06)	116 (65.54)	0.75	1612 (39.28)	1311 (39.48)	262 (37.70)	0.68

Arterial blood pressure, mm Hg^d^	94.06 ± 12.87	94.28 ± 12.61	92.98 ± 12.76	0.46	87.83 ± 11.08	87.55 ± 11.38	87.24 ± 11.05	0.32

Diabetes Present^e^	283 (25.75)	258 (27.95)	58 (32.77)	0.12	534 (13.01)	536 (16.14)	136 (19.57)	<0.00001

Glucose, mg/dL	119.74 ± 54.11	121.25 ± 57.38	128.02 ± 61.89	0.19	105.80 ± 31.34	108.5 ± 35.47	111.18 ± 36.60	0.00002

Triglycerides, mg/dL	115.99 ± 72.35	113.05 ± 60.12	113.64 ± 59.79	0.60	150.57 ± 91.84	149.22 ± 91.66	151.24 ± 116.54	0.78

HDL-C, mg/dL	55.73 ± 18.84	54.78 ± 17.82	53.90 ± 18.83	0.33	51.08 ± 17.70	51.82 ± 18.54	50.20 ± 17.11	0.05

LDL-C, mg/dL	127.88 ± 36.20	129.15 ± 37.24	130.26 ± 37.15	0.61	126.99 ± 33.10	126.56 ± 34.90	127.03 ± 33.21	0.85

Total Cholesterol, mg/dL	206.43 ± 39.13	206.45 ± 38.71	206.89 ± 39.96	0.99	207.90 ± 36.98	207.94 ± 37.95	206.36 ± 35.65	0.57

Data are means ± SD or *n *(%) unless otherwise indicated. Abbreviations: HDL-C, high density lipoprotein cholesterol; LDL-C, low density lipoprotein cholesterol. ^a^*P *value is based on ANOVA (continuous) and χ^2 ^(categorical), comparing differences for individual characteristic across genotypes; ^b^obesity was defined as body mass index ≥30 kg/m^2^; ^c^hypertension was defined as systolic blood pressure ≥140 mmHg or diastolic blood pressure ≥90 mmHg or a history of anti-hypertension medication use; ^d^mean arterial blood pressure was defined as one-third of systolic blood pressure plus two-thirds of diastolic blood pressure; ^e^diabetes was defined as fasting serum glucose levels of at least 7.0 mmol/L (126 mg/dl), nonfasting glucose levels of at least 11.1 mmol/L (200 mg/dl), current use of hypoglycemic medications, or a self-reported physician diagnosis of diabetes.

The associations between retinal lesions and rs7903146 are presented in Table 2. The heterozygous CT-genotype and homozygous TT-genotype individuals had a slightly higher prevalence of retinal lesions when compared with CC-genotype individuals in both races except for AV nicking in Caucasians. Assuming an additive mode of inheritance, the rs7903146 T allele was marginally significantly associated with prevalent focal arteriolar narrowing in Caucasians [OR_CT vs. CC _(95% CIs) = 1.11 (1.00, 1.23); OR_TT vs. CC _(95% CIs) = 1.23 (1.00, 1.51); *P *= 0.05], but not in African American participants [OR_CT vs. CC _(95% CIs) = 1.10 (0.88, 1.36); OR_TT vs. CC _(95% CIs) = 1.20 (0.78, 1.85); *P *= 0.40] (Table 2). No significant associations were noted for AV nicking, retinopathy, or retinal arteriolar or venular diameters (CRAE, CRVE) with rs7903146 (Table 3).

**Table 2:** Retinal lesions by *TCF7L2 *rs7903146 genotype, by race

		African American				Caucasian			
			
Retinal Lesion		*n*	No. with Lesion (%)	**OR (95% CI)**^**a**^	***P *value**^**b**^	*n*	No. with Lesion (%)	**OR (95% CI)**^**a**^	***P *value**^**b**^
AV nicking	CC	1083	179 (16.53)	1.00	0.24	4058	585 (14.42)	1.00	0.58
	
	CT	915	156 (17.05)	1.12 (0.93, 1.35)		3286	433 (13.18)	1.03 (0.93, 1.14)	
	
	TT	174	33 (18.97)	1.26 (0.86, 1.83)		689	106 (15.38)	1.06 (0.86, 1.30)	

Focal arteriolar narrowing	CC	1076	136 (12.64)	1.00	0.40	4041	598 (14.80)	1.00	0.05
	
	CT	912	120 (13.16)	1.10 (0.88, 1.36)		3268	543 (16.62)	1.11 (1.00, 1.23)	
	
	TT	173	26 (15.03)	1.20 (0.78, 1.85)		688	104 (15.12)	1.23 (1.00, 1.51)	

Retinopathy	CC	1099	138 (12.56)	1.00	0.36	4105	236 (5.75)	1.00	0.27
	
	CT	923	128 (13.87)	1.10 (0.90, 1.35)		3321	205 (6.17)	1.09 (0.94, 1.26)	
	
	TT	177	24 (13.56)	1.21 (0.81, 1.81)		695	45 (6.47)	1.18 (0.88, 1.58)	

Abbreviations: AV, arteriovenous; CI, confidence interval; OR, odds ratio. ^a^Adjusted for age, study center, gender, current smoking (yes/no), obesity, total serum cholesterol, total serum triglycerides, mean arterial blood pressure, hypertension, and antihypertensive medication; ^b^*P *value for OR in the log additive genetic model.

**Table 3:** Mean retinal vessel calibers (CRAE/CRVE) by *TCF7L2 *rs7903146 genotype, by race

		African American			Caucasian		
			
Retinal Vessel Index	Geno-type	*n*	**Multivariate Adjusted**^**a**^	***P *value**^**b**^	*n*	**Multivariate Adjusted**^**a**^	***P *value**^**b**^
Mean retinal arteriolar diameter (95% CI), μm	CC	1090	161.61 (160.07, 163.15)	0.49	4096	163.25 (162.42, 164.08)	0.24
	
	CT	916	161.94 (160.40, 163.48)		3312	162.97 (162.16, 163.79)	
	
	TT	177	162.27 (160.24, 164.29)		694	162.69 (161.65, 163.73)	

Mean retinal venular diameter (95% CI), μm	CC	1090	200.36 (198.79, 201.94)	0.32	4096	197.52 (196.69, 198.35)	0.94
	
	CT	916	199.86 (198.28, 201.45)		3312	197.54 (196.72, 198.36)	
	
	TT	177	199.36 (197.25, 201.47)		694	197.56 (196.51, 198.60)	

Abbreviations: CI, confidence interval; CRAE, central retinal artery equivalent; CRVE, central retinal venular equivalent.

^a^Adjusted for age, study center, gender, current smoking (yes/no), obesity, total serum cholesterol, total serum triglycerides, mean arterial blood pressure, hypertension, antihypertensive medication, and CRAE (when the outcome is CRVE)/CRVE (when the outcome is CRAE); ^b^*P *value for 1 degree freedom test of association between vessel calibers and rs7903146 under the log additive genetic model.

Hypertension and diabetes were important effect measure modifiers for focal arteriolar narrowing in Caucasians [multiplicative *P *= 0.03 (hypertension), *P *= 0.04 (diabetes); additive ICR = 0.41 and *P *= 0.006 (hypertension), ICR = -0.29 and *P *= 0.04 (diabetes)], but not in African American participants (*P *> 0.05). When stratified by hypertension or diabetes status, *TCF7L2 *rs7903146 was significantly associated with an increased odds of focal arteriolar narrowing in Caucasian individuals, however only among those with hypertension or without diabetes (Table 4); no associations were noted in African American participants (data not shown). Our analysis in Caucasian individuals with hypertension AND without diabetes indicated that *TCF7L2 *rs7903146 was associated with focal arteriolar narrowing [OR_CT vs. CC _(95% CIs) = 1.40 (1.19, 1.64); OR_TT vs. CC _(95% CIs) = 1.96 (1.43, 2.68); *P *< 0.0001], which is consistent with our interaction analyses. No significant interactions with hypertension or diabetes were observed for other retinal lesions and retinal vessel calibers (CRAE, CRVE).

**Table 4:** Retinal Lesions and *TCF7L2 *rs7903146 genotype by hypertension or diabetes status in Caucasians

		With Hypertension			Without Hypertension			With Diabetes			Without Diabetes		
					
Retinal Lesion		*n*	No. with Lesion (%)	**OR (95% CI)**^**a**^	*n*	No. with Lesion (%)	**OR (95% CI)**^**a**^	*n*	No. with Lesion (%)	**OR (95% CI)**^**a**^	*n*	No. with Lesion (%)	**OR (95% CI)**^**a**^
AV nicking	CC	1595	280 (17.55)	1.00	2462	305 (12.39)	1.00	525	89 (16.95)	1.00	3533	496 (14.04)	1.00
	
	CT	1297	222 (17.12)	1.18 (1.02, 1.36)	1989	211 (10.61)	0.91 (0.78, 1.05)	525	88 (16.76)	0.99 (0.78, 1.26)	2761	345 (12.50)	1.04 (0.92, 1.16)
	
	TT	259	60 (23.17)	1.39 (1.04, 1.86)	430	46 (10.70)	0.83 (0.61, 1.11)	134	21 (15.67)	0.98 (0.61, 1.58)	555	85 (15.32)	1.07 (0.85, 1.35)

*P *value^b^				0.03			0.21			0.94			0.56

Focal arteriolar narrowing	CC	1584	330 (20.83)	1.00	2456	268 (10.91)	1.00	519	88 (16.96)	1.00	3522	510 (14.48)	1.00
	
	CT	1290	320 (24.81)	1.25 (1.09, 1.44)	1978	223 (11.27)	0.96 (0.82, 1.12)	526	91 (17.30)	0.85 (0.65, 1.11)	2742	452 (16.48)	1.18 (1.06, 1.32)
	
	TT	259	67 (25.87)	1.56 (1.18, 2.06)	429	37 (8.62)	0.92 (0.68, 1.25)	136	15 (11.03)	0.73 (0.42, 1.24)	552	89 (16.12)	1.40 (1.12, 1.75)

*P *value^b^				**0.002**			0.59			0.24			**0.003**

Retinopathy	CC	1612	136 (8.44)	1.00	2492	100 (4.01)	1.00	534	78 (14.61)	1.00	3571	158 (4.42)	1.00
	
	CT	1311	112 (8.54)	1.04 (0.85, 1.26)	2010	93 (4.63)	1.12 (0.90, 1.39)	536	76 (14.18)	1.05 (0.82, 1.34)	2785	129 (4.63)	1.01 (0.84, 1.22)
	
	TT	262	23 (8.78)	1.08 (0.73, 1.60)	433	22 (5.08)	1.26 (0.81, 1.94)	136	23 (16.91)	1.10 (0.67, 1.8)	559	22 (3.94)	1.02 (0.70, 1.48)

*P *value^b^				0.71			0.31			0.71			0.91

Abbreviations: AV, arteriovenous; CI, confidence interval; OR, odds ratio.

^a^Adjusted for age, study center, gender, current smoking (yes/no), obesity, total serum cholesterol, total serum triglycerides, mean arterial blood pressure, hypertension (when stratified by diabetes), and antihypertensive medication; ^b^*P *value for OR in the log additive genetic model.

## Discussion

Our study reports on the association between the *TCF7L2 *rs7903146 polymorphism and retinal microvascular lesions and retinal vascular caliber in a middle-aged African-American and Caucasian population. No associations were noted except for focal arteriolar narrowing in Caucasians. The *TCF7L2 *rs7903146 was significantly associated with a greater frequency of focal arteriolar narrowing among Caucasians with hypertension or without diabetes, but not among those without hypertension or with diabetes, suggesting an interaction between *TCF7L2 *variants and hypertension and diabetes status in Caucasians.

To our knowledge, there are few studies for direct comparison. An earlier case-control study in a French population reported the lack of an association with severe retinopathy (effect estimates not reported) [10], which is consistent with our findings on retinopathy in Caucasians. The InCHIANTI study of elderly Europeans reported an association with diabetic retinopathy in 127 persons with diabetes. However the number of participants with diabetic retinopathy was very small (*n *= 12) and results were not statistically significant [11]. Notably, these two studies did not report the definition for retinopathy used, which may differ from ours.

We observed an association between *TCF7L2 *rs7903146 and focal arteriolar narrowing in Caucasians, but not in African Americans. The lack of association in the African American examinees could reflect confounding by unmeasured covariates that are differentially distributed in African American and Caucasian participants, which warrants further investigation. More likely however, the limited power to detect such a modest effect in the African American sample (calculated as 26% for a relative risk of 1.15) may explain our findings (Additional file 1, **Figure S1**). The latter is supported by the observation of very similar effect size estimates between African American and Caucasian participants, and therefore warrants further study in additional African American populations.

It is not known why *TCF7L2 *rs7903146 was associated with retinal focal arteriolar narrowing. To determine whether the effect of *TCF7L2 *rs7903146 on focal arteriolar narrowing was due to hyperglycemia, we further adjusted for fasting glucose values in the models, but no attenuation of genetic effects were noted. It is possible that the *TCF7L2 *rs7903146 variant may be related to focal arteriolar narrowing not through its effect on diabetes but through other, retinal-specific mechanisms (i.e. pleiotropic effects). The Wnt/β-catenin/T-cell factor (TCF) (canonical) signaling pathway may inhibit the adipogenic differentiation of pericytes (a contractile cell in small retinal arterioles), which may have a later effect in regulating retinal microvascular function. This pathway also regulates vascular smooth muscle cell proliferation, suggesting that it may be involved in intimal thickening [23]. Prolonged exposure to elevated blood pressure may lead to retinal vessel vasospasm, intimal thickening, medial hyperplasia and arteriosclerosis manifesting as either generalized or focal arteriolar narrowing [24]. However, we found only a relation with focal and not generalized arteriolar narrowing as measured by CRAE and biological mechanisms remain speculative.

An alternate explanation of our positive findings could be chance considering the large number of comparisons made in assessing association in the context of possible effect modification. To minimize the impact of the multiple tests we could apply a crude Bonferroni correction (five phenotypes in the context of multiple strata defined by diabetes, hypertension, combined diabetes and hypertension grouping, and the full sample N = 30), noting that such an approach is an over-correction because many of the analytic runs assessed the same dependent variable. If such a correction were applied, most of the results reported in this paper would not be statistically significant except in the subgroup with hypertension AND without diabetes.

Our study has notable strengths, including a large, population-based cohort, standardized assessment of retinal photographs, and detailed information on a variety of risk factors. To our knowledge, this is the first population-based study that systematically examines the association between *TCF7L2 *rs7903146 and retinal microvascular lesions and caliber in middle-aged African Americans and Caucasians.

Several important limitations also deserve mention. First, grading was performed from a single 45° fundus photograph that was taken through a nonpharmacologically dilated pupil. This can underestimate the prevalence of retinal microvascular lesions, which could have biased the results toward the null. Second, we found that the *TCF7L2 *rs7903146 is related to higher risk of retinal AV nicking only in Caucasians who had hypertension (*P *= 0.03). This association could have arisen by chance; the pathophysiology underlying any relationship between AV nicking and rs7903146 has not been established. Third, as diabetes and fasting glucose values are plausibly intermediate variables between *TCF7L2 *and retinal phenotypes, our analyses conditional on diabetes/fasting glucose values need to be interpreted with caution as this method may introduce confounding [25]. Fourth, as polymorphisms within *TCF7L2 *possibly impair the glucagon like peptide-1 induced insulin secretion [9], which in turn could lead to a lower postprandial insulin secretion, we might expect to see a stronger effect among patients with impaired glucose tolerance. However, the oral glucose tolerance test was not performed at visit 3 when the fundus photographs were taken. Finally, our samples of African American and diabetic Caucasians are limited to 2,199 and 1,206 examinees, respectively, thus true associations between retinal lesions and the *TCF7L2 *variant could have been missed in these subpopulations. Replication of our findings in other large, population-based studies could help better elucidate these relationships.

## Conclusions

In summary, *TCF7L2 *rs7903146 is not consistently associated with retinal microvascular signs. However, our study is the first to report an association between the *TCF7L2 *rs7903146 polymorphism and focal arteriolar narrowing in Caucasians with hypertension or without diabetes. No significant associations were noted for other retinal microvascular signs in either race group. Other large, population-based studies are needed to confirm our findings.

## Competing interests

CJG is an employee and shareholder of Merck & Co., Inc.

## Authors' contributions

Study design and conduct: YY, KEN, RK, GH, CJG, BEK, KMR, SLW, FLB; collection of data: KEN, RK, GH, BEK, EB; analysis of data: YY, KEN, CJG; interpretation of data: YY, KEN, RK, CJG, EML, AK; preparation of the manuscript: YY, KEN. All authors read and approved the final manuscript.

## Pre-publication history

The pre-publication history for this paper can be accessed here:

http://www.biomedcentral.com/1472-6823/10/9/prepub

## Supplementary Material

Additional file 1 **Power Analysis for the estimation of association between *TCF7L2 *rs7903146 and retinal microvasculature in the ARIC Study**. Additional file 1 contains three supplementary figures on the power analyses performed for the estimation of association between *TCF7L2 *rs7903146 and retinal microvasculature in the ARIC Study. Click here for file
