# Supplementary material for: The transcription factor 7-like 2 (TCF7L2) polymorphism may be associated with focal arteriolar narrowing in Caucasians with hypertension or without diabetes: the ARIC Study
Source: BMC Endocr Disord. 2010 May 17;10:9. doi: 10.1186/1472-6823-10-9 (PMC2879252; doi:10.1186/1472-6823-10-9)
Supplement: Additional file 1 — Power Analysis for the estimation of association between TCF7L2 rs7903146 and retinal microvasculature in the ARIC Study. Additional file 1 contains three supplementary figures on the power analyses performed for the estimation of association between TCF7L2 rs7903146 and retinal microvasculature in the ARIC Study. [file 1472-6823-10-9-S1.DOC]

**Additional file 1**

**Title: Power Analysis for the estimation of association between *TCF7L2* rs7903146 and retinal microvasculature in the ARIC Study.**

Power analyses were performed using QUANTO 1.2 assuming an unmatched case-control study design. Analysis was performed separately for Caucasians and African-Americans, assuming a log-additive model and a two-sided p value of 0.05.

Supplementary Figure 1. Power to detect an odds ratio for retinal microvascular lesions (retinopathy, focal narrowing, A/V nicking) comparing rs7903146 genotype CT vs. CC in the African American and Caucasian ARIC subpopulations assuming a log-additive model, a two-sided statistical test, and a type I error of α = 0.05.

Supplementary Figure 2. Power to detect an odds ratio for retinal microvascular lesions (retinopathy, focal narrowing, A/V nicking) comparing rs7903146 genotype CT vs. CC in the Caucasian ARIC subpopulations by hypertension or diabetes status, assuming a log-additive model, a two-sided statistical test, and a type I error of α = 0.05.

Supplementary Figure 3. Power to detect an averaged difference (µm) in retinal microvascular caliber (CRAE, CRVE) comparing rs7903146 genotype CT vs. CC in the African American and Caucasian ARIC subpopulations, assuming a log-additive model, a two-sided statistical test, and a type I error of α = 0.05.
